# Neonatal Tooth in Fraternal Twins: A Case Report

**DOI:** 10.5005/jp-journals-10005-1028

**Published:** 2009-08-26

**Authors:** Prabhakar AR, Ravi GR, Raju OS, Ameet J Kurthukoti, Shubha AB

**Affiliations:** 1 Professor and Head, Department of Pedodontics and Preventive Dentistry, Bapuji Dental College and Hospital Davangere-577004, Karnataka, India; 2 Ex-Postgraduate Student, Department of Pedodontics and Preventive Dentistry, Bapuji Dental College and Hospital Davangere-577004, Karnataka, India; 3 Professor, Department of Pedodontics and Preventive Dentistry, Bapuji Dental College and Hospital Davangere-577004, Karnataka, India; 4 Reader, Department of Pedodontics and Preventive Dentistry, Bapuji Dental College and Hospital Davangere-577004, Karnataka, India; 5 Assistant Professor, Department of Pedodontics and Preventive Dentistry, Bapuji Dental College and Hospital Davangere-577004, Karnataka, India

**Keywords:** Neonatal teeth, fetal teeth, fraternal twins.

## Abstract

Eruption of teeth at or immediately after birth is a relatively rare phenomenon. This condition has been the subject of curiosity and study since the time of beginning of beliefs and assumptions. The present case report accentuates the occurrence of neonatal teeth in twins; fraternal twins in particular which is rarest of its kind.

## INTRODUCTION

Eruption of teeth at or immediately after birth is a relatively rare phenomenon.^1^ According to Masler and Savara (1950),^2^ natal teeth are defined as any teeth present at birth, while neonatal teeth are those erupting in the first month of life.^3^,^4^

These prematurely erupted primary teeth have also been described in the literature as ‘congenital teeth’, ‘fetal teeth’ or ‘dentition praecox.‘ Neonatal teeth often present with hypoplastic enamel and underdeveloped roots with resultant mobility, however, such teeth also should be further classified according to their degree of maturity.^1^,^4^ A mature natal or neonatal tooth is one that exhibits normal development, hence has a relatively good prognosis; while the term immature natal and neonatal tooth implies defective development and poor prognosis for retention.

Various reports have been documented in the literature about the occurrence of natal and neonatal teeth. On the contrary, their incidence in twins is the rarest of its kind as there is no citation of the same in the literature. The purpose of this report is to highlight the occurrence of neonatal tooth in twins, probably the first of its kind to be reported.

Twins are two offspring born at the same time. There are two major types of twins: monozygotic and dizygotic. Monozygotic twins are identical and may be separate or conjoined where as dizygotic twins are usually nonidentical and are also called fraternal twins.^5^ We are presenting the case report of neonatal tooth in fraternal twins.

## LITERATURE REVIEW

Neonatal teeth have been a subject of curiosity and study since the time it was first documented in 59 BC. This led to the beginning of beliefs and assumptions about neonatal teeth. In countries like Poland, India and among African tribes the children with these teeth have been killed soon after the birth, as it was believed to bring misfortune.^4^,^6^ In England, it was thought that these babies would become great soldiers whereas in France and Italy this condition would guarantee the conquest of world. Famous personalities like Zoroaster, Hannibal, Louis XIV, Mazarin, Richelieu, Mirabeau, Richard III and Napoleon were born with these teeth.^4^,^6^

Natal teeth are encountered more often than neonatal teeth in an approximate ratio of 3:1 with more predilections in females, but two recent studies have shown no significant differences.^3^,^4^,^6^-^8^ The incidence of natal teeth is usually quoted in the range of 1:2000 to 1:3500 live births. Natal and neonatal teeth present almost exclusively in the mandibular incisor region, probably because these teeth are normally the first to erupt.^3^,^8^ According to Bodenhoff’s study of natal and neonatal teeth, 85% are mandibular incisors, 11% maxillary incisors, 3% are mandibular canines and molars, and only 1% are maxillary canine or molars.^8^

Over the years there have been many postulations regarding the cause of premature eruption including hypovitaminosis, hormonal stimulation, trauma, febrile states, pyelitis and syphilis, but a cause and effect relationship has not yet been established. The current concept suggests that, natal and neonatal teeth are attributed to a superficial position of the developing tooth germ, which predisposes the tooth to erupt early.^4^,^6^,^8^ Many investigators have reported natal and neonatal teeth as a familial trait with a frequency ranging from 8 to 62% which reflects the hereditary factor.^10^,^12^ Bodenhoff and Gorlin^11^ reported a familial association in 14.5 percent of cases while, Kates, Needleman and Holmes^7^ found a positive family history in 7 out of 38 cases of natal and neonatal teeth. Histological studies have shown that despite normal structure of the enamel of natal and neonatal teeth, early eruption interrupts the mineralization process of enamel.^13^-^15^ Hence, the enamel has often been described as dysplastic or hypomineralized and is prone to wear and discoloration.^9^,^16^

Natal and neonatal teeth may resemble normal primary teeth; but, in many instances, they are poorly developed, small, conical, yellowish, with white hypoplastic enamel and dentin, and with poor or total failure of development of roots.^4^,^17^ The appearance of each natal tooth can be classified in one of the following categories.^17^

- *Category 1:* A shell like crown structure loosely attached to the alveolus by a rim of oral mucosa; no root.
- *Category 2:* A solid crown loosely attached to the alveolus by oral mucosa; little or no root.
- *Category 3:* The incisal edge of the crown just erupted through the oral mucosa.
- *Category 4:* A mucosal swelling with the tooth unerupted but palpable.

It has been recommended that natal teeth of category 1 and 2 are the candidates for extraction if the degree of mobility is more than 2 mm.^17^

The management of natal teeth is dependent on several factors. If the tooth is a supernumerary tooth, then extraction is the treatment of choice. Excessively mobile teeth are usually extracted, due to the risk of exfoliation and subsequent swallowing or inhalation.^2^ A review of the literature, however, reveals that there are no reported cases of aspiration of natal or neonatal teeth. Further, natal and neonatal teeth will exfoliate prematurely, due to inadequate root formation and mobility if left in the mouth.^16^,^18^,^19^

## CASE REPORT

One month old twin female patients reported to the Department of Pedodontics and Preventive Dentistry, Bapuji Dental College and Hospital for the evaluation of tooth present in the mandibular central incisor region. History revealed preterm birth of fraternal twins, (both female) at 32 weeks and the tooth had appeared after 15 days and similar findings were present in the twin. They were the first in the family and there were no dental developmental anomalies reported in the histories of paternal/maternal side. Both were exclusively bottle fed, as the twins had difficulty in feeding and suckling, and also the mother experienced discomfort feeding them. Further the mother was worried that the loose tooth might get dislodged and aspirated. Although they were bottle fed, there were no signs of weight loss and the general health was normal.

**Figs 1A and B:**
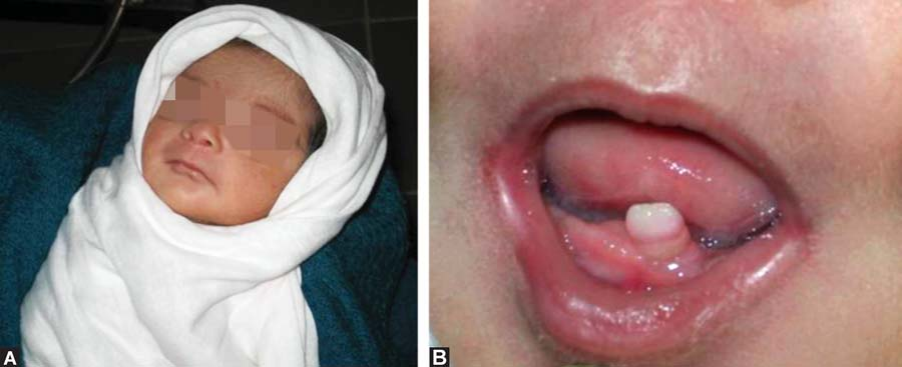
(A) Clinical photograph of the twin (B) showing the neonatal tooth

**Figs 2A and B:**
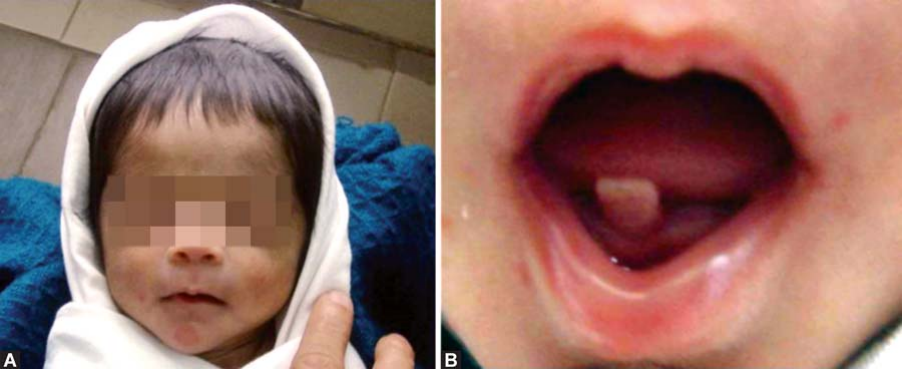
(A)Clinical photograph of the twin (B) showing the neonatal tooth

Intraoral examination revealed the presence of fully erupted neonatal tooth, loosely attached to the alveolus and exhibited no root formation in both the twins. However, in one infant it was observed at mandibular primary left central incisor position with thin enamel and exhibiting grade II mobility (Figs 1A and B); while in other at the mandibular primary right central incisor position, exhibiting grade I mobility (Figs 2A and B).

**Figs 3A and B:**
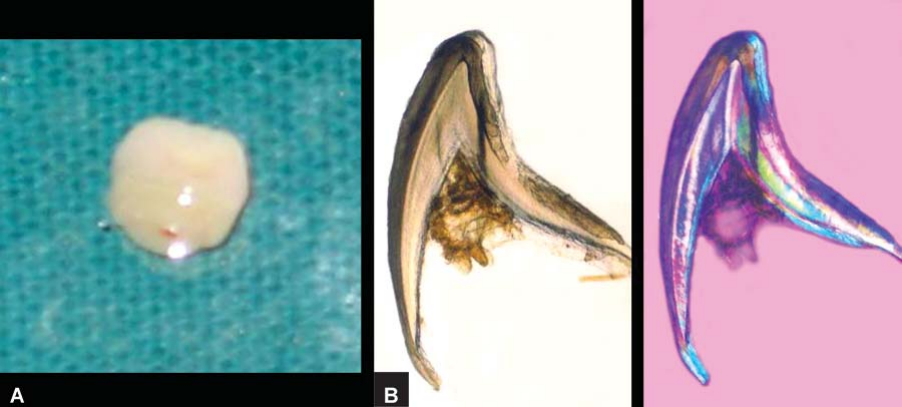
(A) Extracted neonatal tooth of the twin (B) and its ground sections under day-light and polarized-light microscopic examination exhibiting formation of enamel, dentin and pulp

**Figs 4A and B:**
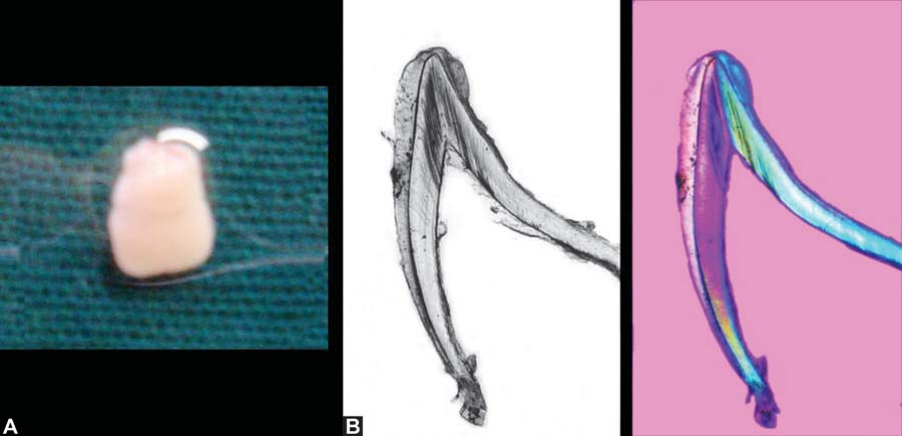
(A) Extracted neonatal tooth of the twin (B) and its ground sections under day-light and polarized-light microscopic examination exhibiting formation of enamel, dentin and pulp

Extraction was chosen as treatment of choice as both the teeth were belonging to category 2.^17^ Prophylactic administration of vitamin K was done one hour prior to the procedure after obtaining the consent from the neonatologist. The procedure was done under topical local anesthesia, which both the patients tolerated well followed by curettage. Curettage was performed to remove the epithelial remnants completely. Following extraction, the teeth were longitudinally sectioned and were then subjected to daylight and polarized-light microscopic examination which revealed the formation of enamel, dentin and pulp (Figs 3A to 4B). The twins were reviewed after one month and the extraction site had fully resolved and they were taking the feed normally.

## DISCUSSION

Everyone everywhere seems fascinated with twins; it doesn’t seem to matter whether they are boys or girls or both. But one thing frequently inquired about is if they are identical or fraternal. Simply put, identical twins are identical in their genetics and fraternal twins are no more alike that any brother and sister.^20^ Identical twins must always be the same gender while fraternal twins can be boys, girls or both. The occurrence of neonatal teeth in twins (fraternal twins in particular) is probably being reported for the first time. In our case though the tooth located in the central incisor region its position varied and this variation could always be seen in nonidentical twins.

Appropriate and timely management of natal teeth is important depending upon the clinical situation. Most prematurely erupted teeth (immature type) are hypermobile because of the limited root development. Neonatal teeth are usually less mobile as compared to natal teeth.^3^,^21^ In some cases the tooth may become mobile to the extent, which may require extraction so as to avoid displacement or aspiration. Also, in exceptionally rare cases in which the sharp incisal edge of the tooth may cause trauma to the surrounding soft tissue, the tooth may have to be extracted. If the tooth is not causing any difficulty to the infant or mother it should be left alone and the importance of tooth in the growth should be explained to the parents. For teeth causing trauma the preferred, conservative treatment consists of smoothing rough incisal edges, or placing round, smooth composites over the incisal edges. A retained natal or neonatal tooth may also cause difficulty for a mother to feed her child. If breast feeding is difficult initially, the use of breast pumps and storing of milk is recommended. However, the infant soon gets conditioned not to ‘bite’ during suckling. It seems that the infant senses the discomfort and learns to avoid causing it. Generally the gingival tissues surrounding natal teeth are normal; occasionally they are, however edematous and hemorrhagic. It has been recommended that the inflamed gingival tissue around the teeth can be controlled by applying chlorhexidine gluconate gel three times a day.^22^

In the present case, the neonatal tooth in the twins was mobile and they were having difficulty in feeding and suckling. So, it was decided to extract the neonatal tooth followed by curettage to allow rapid healing. Extraction of a natal tooth should not present significant difficulties; the underdeveloped cells of dental papilla and those of Hertwig’s root sheath are easily detached from the calcified part of the tooth. However, these cells may remain in the alveolus, and can later continue to develop into tooth like structure.^23^,^24^ This occurs in 9.1% of the children and in few of them it may result in an alveolar abscess.^22^ Hence, extraction of natal teeth and neonatal teeth should be followed by curettage of the socket to prevent continued development of the cells of the dental papilla.

Delaying surgical procedures on newborns until after 10th postpartum day is no longer considered routinely necessary because of prophylactic administration of vitamin K as a standard procedure in most hospitals.^25^ If necessary, hemostasis may be enhanced by using topical hemostatic agents in combination with direct pressure.

As neonatal and natal teeth may present with different clinical manifestations, each case must be evaluated independently and sound clinical judgment must be used to decide whether to retain or extract the tooth in question.
